# Supplementary material for: Melanopsin-mediated image statistics from natural and human-made environments
Source: Sci Rep. 2025 Aug 15;15:29965. doi: 10.1038/s41598-025-15981-y (PMC12356983; doi:10.1038/s41598-025-15981-y)
Supplement: Supplementary file 1 — Supplementary Material 1 [file 41598_2025_15981_MOESM1_ESM.docx]

Supplementary information

“**Melanopsin-mediated image statistics in natural and human-made environments**”

Pablo Barrionuevo & Francisco Diaz Barrancas

**Table S1. Name and sources of the hyperspectral images used in this study.**

| Environment | Name | Associated article | Scene | Obtained from |
| --- | --- | --- | --- | --- |
| Natural | Bom_Jesus_Bush | Nascimento, Amano & Foster (2016)^1^ | 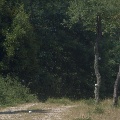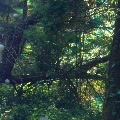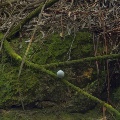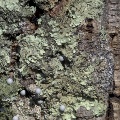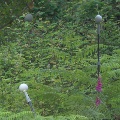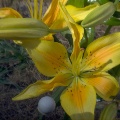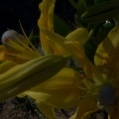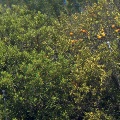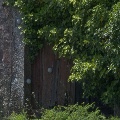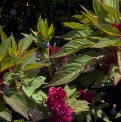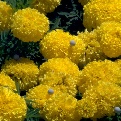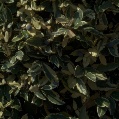 | A |
| Natural | Bom_Jesus_Marigold | Nascimento, Amano & Foster (2016) ^1^ |  | A |
| Natural | Bom_Jesus_Red_flower | Nascimento, Amano & Foster (2016) ^1^ |  | A |
| Natural | Bom_Jesus_Ruin | Nascimento, Amano & Foster (2016) ^1^ |  | A |
| Natural | Gualtar_Orange_Trees | Nascimento, Amano & Foster (2016) ^1^ |  | A |
| Natural | Lillies_Closeup | Nascimento, Amano & Foster (2016) ^1^ |  | A |
| Natural | Lilly_Closeup | Nascimento, Amano & Foster (2016) ^1^ |  | A |
| Natural | Ruivaes_Fern | Nascimento, Amano & Foster (2016) ^1^ |  | A |
| Natural | Ruivaes_Ruin | Nascimento, Amano & Foster (2016) ^1^ | 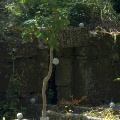 | A |
| Natural | Sameiro_Bark | Nascimento, Amano & Foster (2016) ^1^ |  | A |
| Natural | Sameiro_Branch | Nascimento, Amano & Foster (2016) ^1^ |  | A |
| Natural | Sameiro_Forest | Nascimento, Amano & Foster (2016) ^1^ |  | A |
| Natural | Sameiro_Glade | Nascimento, Amano & Foster (2016) ^1^ |  | A |

| Environment | Name | Associated article | Scene | Obtained from |
| --- | --- | --- | --- | --- |
| Natural | Sameiro_Leaves | Nascimento, Amano & Foster (2016) ^1^ |  | A |
| Natural | Sameiro_Trees | Nascimento, Amano & Foster (2016) ^1^ |  | A |
| Natural | Sete_Fontes_Rock | Nascimento, Amano & Foster (2016) ^1^ |  | A |
| Natural | Tibaes_Garden | Nascimento, Amano & Foster (2016) ^1^ |  | A |
| Natural | Yellow_Rose | Nascimento, Amano & Foster (2016) ^1^ |  | A |
| Natural | levada_1411 | Foster, Amano & Nascimento (2016)^2^ |  | B |
| Natural | nogueiro_1441 | Foster, Amano & Nascimento (2016) ^2^ |  | B |
| Natural | sete_fontes_1438 | Foster, Amano & Nascimento (2016) ^2^ |  | B |
| Human-made | Braga_Grafitti | Nascimento, Amano & Foster (2016) ^1^ |  | A |
| Human-made | Gualtar_Columns | Nascimento, Amano & Foster (2016) ^1^ |  | A |
| Human-made | Gualtar_Villa | Nascimento, Amano & Foster (2016) ^1^ |  | A |
| Human-made | Souto_Farm_Barn | Nascimento, Amano & Foster (2016) ^1^ |  | A |
| Human-made | Souto_Roof_Tiles | Nascimento, Amano & Foster (2016) ^1^ |  | A |
| Human-made | Souto_Wood_Pile | Nascimento, Amano & Foster (2016) ^1^ | 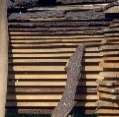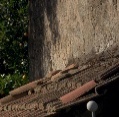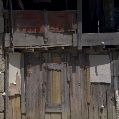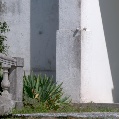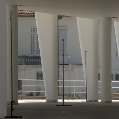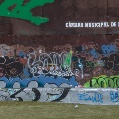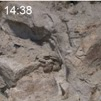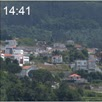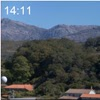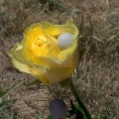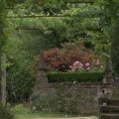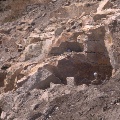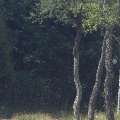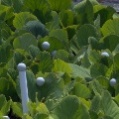 | A |

| Environment | Name | Associated article | Scene | Obtained from |
| --- | --- | --- | --- | --- |
| Human-made | Tenoes_Wall | Nascimento, Amano & Foster (2016) ^1^ |  | A |
| Human-made | Tenoes_Wall_Closeup | Nascimento, Amano & Foster (2016) ^1^ |  | A |
| Human-made | Tibaes_Corridor | Nascimento, Amano & Foster (2016) ^1^ |  | A |
| Human-made | Gualtar_Steps | Nascimento, Amano & Foster (2016) ^1^ | 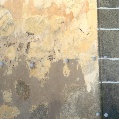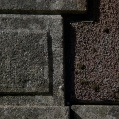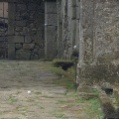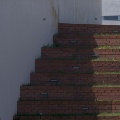 | A |

A) <https://sites.google.com/view/sergionascimento/home/scientific-data/hyperspectral-images-for-spatial-distribution-of-local-illumination-2015>. B) <https://sites.google.com/view/sergionascimento/home/scientific-data/time-lapse-hyperspectral-radiance-images-2015>.

**Table S2. Summary of the statistics values from t-tests of natural versus human-made environments.**

|  | Excitation | | | | Contrast | | | |
| --- | --- | --- | --- | --- | --- | --- | --- | --- |
|  | mean | | variance | | mean | | variance | |
|  | t | p | f | p | t | p | f | p |
| Melanopsin | -5.390 | 1.17E-07 | 0.890 | 0.439 | -1.095 | 0.274 | 1.000 | 0.999 |
| ipRGC_1_ | -4.299 | 2.14E-05 | 1.015 | 0.938 | -1.235 | 0.217 | 0.931 | 0.592 |
| Luminance | -3.667 | 2.78E-04 | 0.977 | 0.861 | -0.615 | 0.539 | 0.929 | 0.583 |

**Figure S1. Extrapolation of the findings.** To test the generalization of our findings, we used a second set of scenes. The scenes were obtained from a publicly available repository^3,4^. This repository contains images with reflectance values for each pixel and was organized by the same authors of the original set; therefore, many scenes that were used in the original database are in this repository, but without radiance information. We used scenes from this repository that were NOT used in the original analysis. These images were 15 from natural environments (hsi: 2, 4, 7,10, 13, 14, 16, 25, 26, 38, 41, 42, 43, and 44), and 9 from human-made environments (hsi: 1, 6, 21, 31, 32, 33, 34, 36, and 39). This classification was validated with the use of our custom AI-based image classifier. We used the tabulated daylight illuminant of correlated color temperature 6500K to obtain relative radiance values.

From this analysis, we found that human-made environments generated higher values of melanopsin, ipRGC, and luminance than natural environments, in agreement with the original analysis using a different dataset (Fig. S2A-C). We also found that the contrast of luminance was higher than the contrasts of melanopsin and ipRGC1 for natural environments [F(2, 592) = 5.34, p < 0.01], in agreement with our original analysis with a different image set. For human-made environments, as expected, melanopsin and luminance were not different; however, a significant (although slight) difference was found between ipRGCs and luminance (p < 0.05) (See Fig. S2D-E). Furthermore, the difference between melanopsin and luminance contrasts increased at higher contrasts (Fig. S2F-G) in agreement with our original analysis (Fig. 3C-D). In conclusion, our findings are supported by this analysis with a new set of images. Differences between groups are indicated as (*) for p < 0.05, (***) for p < 0.001, (****) for p < 0.0001, and (ns) for no difference.

*
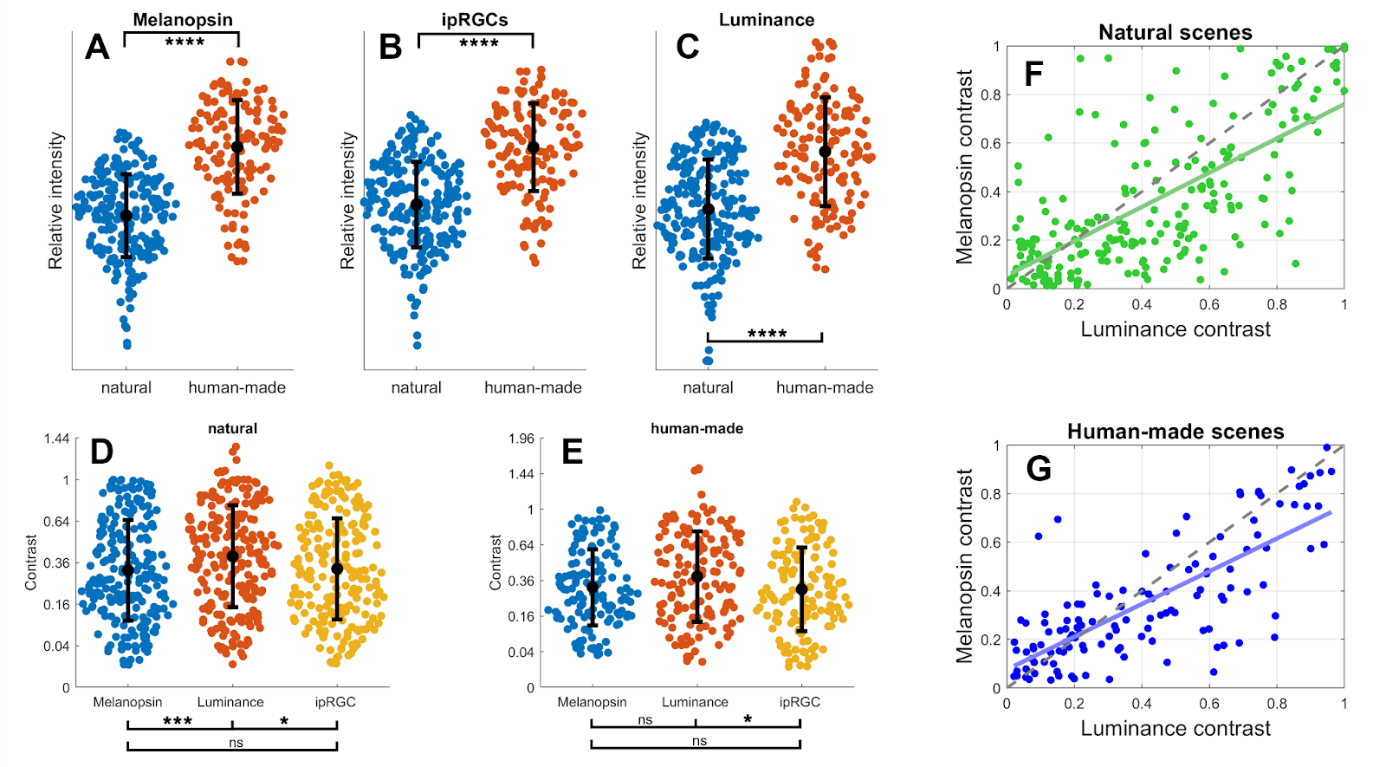
*

**Figure S2. Melanopsin versus luminance contrast for the same field size.** These scatter plots show the high correlation between melanopsin and luminance contrasts in both environments when the same, but not real, receptive field size is used (1.37°). These results showed the importance of using the proper receptive field sizes when comparing luminance and melanopsin signals.

**
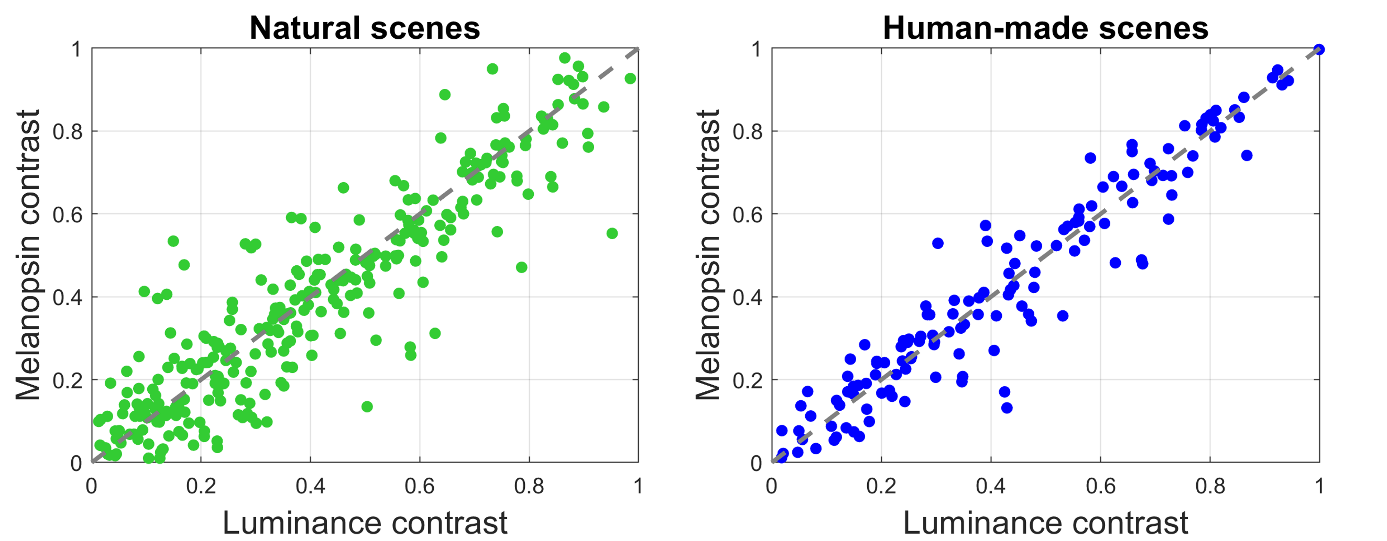
**

**Figure S3. Computation validation.** To validate the computation, we generated artificial hyperspectral scenes. Three of these scenes were generated using the silent substitution method^5,6^, which allows for generting stimulation that selectively stimulates individual or combined photoreceptor excitations. These scenes were divided into sixteen patches; each patch could contain a positive or negative contrast for an intended excitation (luminance or melanopsin). The first scene only differed in luminance (combined L- and M-cone excitation change, while maintaining stable excitation of melanopsin, S-cones, and rods). The second scene only differed in melanopsin excitation (while maintaining stable excitation of rods, L-, M-, and S-cones). The third scene completely produced an equal energy spectrum (no contrast changes for any photoreceptor). The fourth scene replicated the output values of a five-primary photostimulator, which is a device built to selectively stimulate melanopsin in laboratory settings^7^. We computed contrast for these four artificial scenes with the same codes that we used for the main analyses. As expected, our contrast computation generated changes only for the intended excitation. These results validated our computations and ensured non-spurious signals.


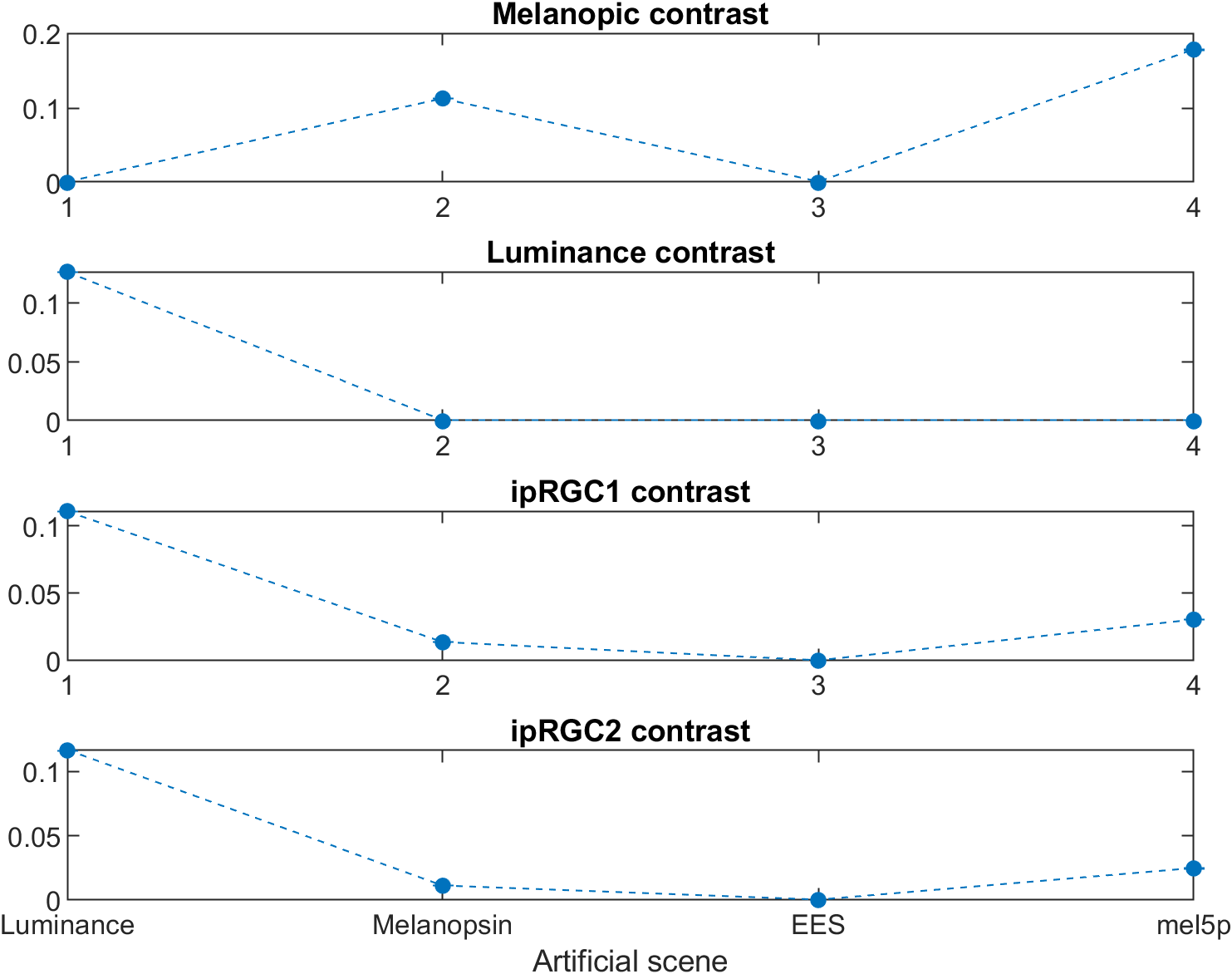


**References**

1. Nascimento, S. M. C., Amano, K. & Foster, D. H. Spatial distributions of local illumination color in natural scenes. *Vision Research* **120**, 39–44 (2016).

2. Foster, D. H., Amano, K. & Nascimento, S. M. C. Time-lapse ratios of cone excitations in natural scenes. *Vision Research* **120**, 45–60 (2016).

3. Foster, D. H. & Reeves, A. Colour constancy failures expected in colourful environments. *Proceedings of the Royal Society B: Biological Sciences* **289**, 20212483 (2022).

4. Foster, D. H., Amano, K. & Nascimento, S. M. C. Fifty hyperspectral reflectance images of outdoor scenes. University of Manchester https://doi.org/10.48420/14877285.v3 (2022).

5. Estévez, O. & Spekreijse, H. The ‘silent substitution’ method in visual research. *Vision Res.* **22**, 681–691 (1982).

6. Barrionuevo, P. A., Sandoval Salinas, M. L. & Fanchini, J. M. Are ipRGCs involved in human color vision? Hints from physiology, psychophysics, and natural image statistics. *Vision Research* **217**, 108378 (2024).

7. Cao, D., Nicandro, N. & Barrionuevo, P. A. A five-primary photostimulator suitable for studying intrinsically photosensitive retinal ganglion cell functions in humans. *J Vis* **15**, 27 (2015).
